# Predictors of mental health deterioration from pre- to post-COVID-19 outbreak

**DOI:** 10.1192/bjo.2022.555

**Published:** 2022-08-30

**Authors:** Nathaly Rius Ottenheim, Kuan-Yu Pan, Almar A. L. Kok, Frederike Jörg, Merijn Eikelenboom, Melany Horsfall, Rob A. Luteijn, Patricia van Oppen, Didi Rhebergen, Robert A. Schoevers, Brenda W. J. H. Penninx, Erik J. Giltay

**Affiliations:** Department of Psychiatry, Leiden University Medical Center, The Netherlands; Department of Psychiatry, Amsterdam Public Health, Amsterdam University Medical Center, Vrije Universiteit, The Netherlands; and Department of Psychiatry, Geestelijke Gezondheidszorg (GGZ) InGeest, The Netherlands; Department of Psychiatry, Amsterdam Public Health, Amsterdam University Medical Center, Vrije Universiteit, The Netherlands; and Department of Psychiatry, GGZ InGeest, The Netherlands; University Center for Psychiatry, University Medical Center Groningen, The Netherlands; and Research Department, GGZ Friesland, The Netherlands; Department of Psychiatry, Amsterdam Public Health, Amsterdam University Medical Center, Vrije Universiteit, The Netherlands; Department of Psychiatry, GGZ InGeest, The Netherlands; and Mental Health Care Institute, GGZ Centraal, The Netherlands; University Center for Psychiatry, University Medical Center Groningen, The Netherlands

**Keywords:** Anxiety disorders, depressive disorders, epidemiology, COVID-19, neuroticism

## Abstract

**Background:**

Mental health was only modestly affected in adults during the early months of the COVID-19 pandemic on the group level, but interpersonal variation was large.

**Aims:**

We aim to investigate potential predictors of the differences in changes in mental health.

**Method:**

Data were aggregated from three Dutch ongoing prospective cohorts with similar methodology for data collection. We included participants with pre-pandemic data gathered during 2006–2016, and who completed online questionnaires at least once during lockdown in The Netherlands between 1 April and 15 May 2020. Sociodemographic, clinical (number of mental health disorders and personality factors) and COVID-19-related variables were analysed as predictors of relative changes in four mental health outcomes (depressive symptoms, anxiety and worry symptoms, and loneliness), using multivariate linear regression analyses.

**Results:**

We included 1517 participants with (*n* = 1181) and without (*n* = 336) mental health disorders. Mean age was 56.1 years (s.d. 13.2), and 64.3% were women. Higher neuroticism predicted increases in all four mental health outcomes, especially for worry (*β* = 0.172, *P* = 0.003). Living alone and female gender predicted increases in depressive symptoms and loneliness (*β* = 0.05–0.08), whereas quarantine and strict adherence with COVID-19 restrictions predicted increases in anxiety and worry symptoms (*β* = 0.07–0.11).Teleworking predicted a decrease in anxiety symptoms (*β* = −0.07) and higher age predicted a decrease in anxiety (*β* = −0.08) and worry symptoms (*β* = −0.10).

**Conclusions:**

Our study showed neuroticism as a robust predictor of adverse changes in mental health, and identified additional sociodemographic and COVID-19-related predictors that explain longitudinal variability in mental health during the COVID-19 pandemic.

The COVID-19 pandemic has led to a profound change in daily routines and social interactions. In The Netherlands, as in other countries, far-reaching pandemic-induced changes, such as travel and social restrictions, were implemented to limit the spread of the virus. People were asked to work from home as much as possible and comply with social distancing, and all large events were cancelled. On 15 March 2020, a so-called ‘intelligent lockdown’ was implemented (www.government.nl/topics/coronavirus-covid-19). All schools and child care facilities were closed (except for children whose parents had an essential occupation), as well as sports and leisure facilities, bars and restaurants. These restrictions had wide-ranging effects, such as social isolation and financial concerns derived from job insecurity.^1^ Understanding how these unintended consequences of the COVID-19 restrictions affect individuals differently depending on their sociodemographic and personality characteristics may shed more light on which groups are at higher risk of mental health deterioration, and so inform policy making.

In a previous study, we found that the severity of psychiatric symptoms after the COVID-19 outbreak was higher in those with premorbid mental health problems; however, surprisingly, it was the healthy controls who reported larger, albeit modest, increases in symptoms compared with pre-COVID-19 levels.^2^ Importantly, we observed large inter-individual variation in change in mental health severity scores. This finding demonstrated a need for a better understanding of which factors best predict adverse changes in mental health, to develop targeted preventive interventions aimed at those most vulnerable. Multiple studies have now been published on the impact of the COVID-19 pandemic on mental health, but most of these studies had methodological design issues, such as cross-sectional designs, absent pre-pandemic data, using different samples when comparing pre- and post-pandemic data, or using limited predictor and outcome measures. A recent systematic review reported a particular vulnerability for detrimental effects of the COVID-19 pandemic in patients with depression and anxiety, and stressed the need to better identify those individuals at the highest risk so as to offer them targeted mental healthcare, and to improve their social support and coping strategies.^3^

## A unique opportunity

In the current study, we aimed to determine which sociodemographic, clinical, personality and COVID-19-related factors were independently associated with a relative change in mental health scores during the first months of the COVID-19 pandemic compared with pre-COVID-19 levels. Combining three large, well-established longitudinal cohorts with similar pre-pandemic data and clinical diagnoses offered us a unique opportunity to overcome some of the methodological limitations of earlier studies.

## Method

### Participants

We combined data from three prospective cohorts that used largely similar methodology for data collection. A more detailed description of these cohorts has been given elsewhere.^2^ Briefly, in April 2020, participants from the Netherlands Study of Depression and Anxiety (NESDA),^4^ Netherlands Study of Depression in Older Persons (NESDO)^5^ and Netherlands Obsessive Compulsive Disorder Association Study (NOCDA)^6^ were asked to participate in follow-up online surveys on their mental health during the COVID-19 pandemic. NESDA is an ongoing longitudinal study examining the development and course of depression and anxiety disorders among people aged 18–65 years with a depression or anxiety disorder (*n* = 2329), biological siblings (*n* = 367) and individuals without a mental health disorder (*n* = 652). Between 2004 and 2007, participants were recruited from the community, primary care and specialised mental healthcare in The Netherlands, and they were followed up at 2, 4, 6 and 9 years. NESDO is a longitudinal study of depression in older people (aged 60–93 years). From 2007 until 2010, 378 individuals with a depressive disorder were recruited through specialised mental healthcare services and 132 individuals without a mental health disorder were recruited from primary care. Face-to-face assessments were done after 2 and 6 years. NOCDA is a longitudinal study in 419 people aged 18–65 years with a lifetime diagnosis of obsessive–compulsive disorder who were recruited from mental healthcare institutions. Baseline assessments were done between 2004 and 2009, and follow-up examinations took place after 2, 4 and 6 years. In NESDA and NESDO, the DSM-IV-based Composite Interview Diagnostic Instrument was used to diagnose mental health disorders; in NOCDA, the Structured Clinical Interview for DSM-IV Axis I disorders was used for diagnosis. Lifetime and current (within the past 6 months) presence of six disorders was assessed at all previous waves in all three cohorts: major depressive disorder, dysthymia, general anxiety disorder, panic disorder, social phobia and agoraphobia. The diagnosis of obsessive–compulsive disorder was added in NOCDA only.

For the current study, participants who had completed the most recent pre-COVID-19 wave, and had given permission to be approached for future studies, were sent an invitation to participate in the COVID-19 online assessments. Of all eligible participants, 1517 (58%) participants provided online informed consent and completed the online questionnaire at least once during the first four waves of data collection between 1 April and 15 May 2020. The online questionnaire was built in Survalyzer, 3000 edition for Windows (Survalyzer, Zürich, Switzerland; https://www.survalyzer.com/). We used the first response per participant in combination with their pre-pandemic data gathered between 2006 and 2016. It is important to note that the final wave had taken place longer than 6 months before the COVID-19 outbreak. The pre-COVID-19 assessment was calculated as the mean of the four main outcomes based on data from the up to four most recent available waves). The authors assert that all procedures contributing to this work comply with the ethical standards of the relevant national and institutional committees on human experimentation and with the Helsinki Declaration of 1975, as revised in 2008. All procedures involving human patients were approved by the Institutional Review Board of the Vrije Universiteit Medical Center, Amsterdam (reference number 2020.166), and adhered to the Declaration of Helsinki. Written informed consent was obtained from all patients.

### Measurements

#### Independent variables

Sociodemographic factors were based on pre-pandemic assessments, including age, gender and education level (categorised as basic (elementary school), intermediate (lower vocational to general secondary education) and higher (college or university)). From the COVID-19 online questionnaire, we included information on living situation (alone or cohabiting), occupation (being an essential worker or not) and whether participants had a house with outdoor space during the lockdown (yes/no). COVID-19 pandemic factors were as follows: having been quarantined in the two weeks before the assessment (yes/no), whether participants were strictly following COVID-19 guidelines (five-point Likert scale), whether they were teleworking or taking care of children at home (yes/no) and whether the pandemic had led to a change in their daily routines (characterised as more telework, taking care of children because of school closure, taking care of a sick family member or other).

We also included pre-COVID-19 clinical information on personality characteristics based on data from previous waves (2007–2009). Because different instruments were used (the 60-item questionnaire NEO-Five Factor Inventory^7^ in NESDA and NESDO, and the 100-item Five Factor Personality Inventory^8^ in NOCDA), these personality scores were standardised before merging the two data-sets. The Big Five personality characteristics were assessed: neuroticism, extraversion, openness to experience, agreeableness and conscientiousness. For clinical factors, we used pre-pandemic data on current (6-month) diagnosis of depression, anxiety and obsessive–compulsive disorders in consecutive waves, to assess the number of mental disorders.

#### Mental health outcomes (dependent variables)

Four validated symptom severity scales were included in the pre-pandemic waves and the COVID-19 questionnaires. For depressive symptoms ,we used the 16-item Quick Inventory of Depressive Symptoms (QIDS);^9^ for anxiety symptoms, we used the 21-item Beck Anxiety Inventory (BAI);^10^ for worry symptoms, we used the 11-item Penn State Worry Questionnaire (PSWQ);^11^ and for loneliness, we used the six-item De Jong Gierveld Loneliness Scale.^12^

#### Missing data

There were no missing data for gender, age, education, living alone or number of mental health disorders. Other variables had some missing data: home with outdoor space (*n* = 1), quarantine in prior 2 weeks (*n* = 1), change of activity upon COVID-19 (*n* = 1), working from home (teleworking) (*n* = 1), taking care of children at home (*n* = 1), essential worker (*n* = 9), strictly following COVID-19 guidelines (*n* = 29), extraversion (*n* = 122), agreeableness (*n* = 128), conscientiousness (*n* = 128), neuroticism (*n* = 251) and openness to experience (*n* = 253). Moreover, changes in outcome variables were missing for the change in depressive symptoms (QIDS; *n* = 147), anxiety symptoms (BAI; *n* = 41), worry symptoms (PSWQ; *n* = 224) and loneliness (De Jong Gierveld Loneliness Scale; *n* = 229). Multiple imputation was applied for missing values.

### Statistical analyses

Data are summarised as numbers (percentages) and means (s.d.), as appropriate. Changes in the four severity scales (i.e. QIDS, BAI, PSWQ, De Jong Gierveld Loneliness Scale) were calculated as the difference between the COVID-19 wave values minus the pre-COVID-19 wave values, which we standardised for the analyses. Histograms were plotted to analyse the distributions of each of the four (unstandardised) delta scores and their change scores.

Next, the 17 putative independent variables were also standardised, so that the standardised beta-coefficients would be directly comparable regarding their relative strengths. Multiple imputation was applied for missing values. The data-set with missing values was copied five times according to Rubin's rule, after which the missing values were replaced with imputed values in each copy of the data-set, taking random variation into account. These imputed data-sets were each analysed, after which the results were pooled and depicted in forest plots for each of the four outcome variables separately. Analyses were done with univariable and multivariable linear regression analysis. In the latter, all independent variables were entered into the model. The delta scores served as the dependent variables, adjusting for the respective baseline severity score. As all variables were standardised, this yielded standardised beta-coefficients, which could be compared with each other to appreciate the relative strengths of each of the associations. We used multiple imputation to deal with missing data, with replacement values for multivariate missing data based on all covariates. The method was based on fully conditional specification, with five multiple imputations per analysis. In subsequent analyses, effect modification by the number of lifetime psychiatric disorders (i.e. as a continuous variable in the interaction term: predictor×number of disorders) was explored for each of the 17 independent variables and each of the four outcome variables, for which the beta-coefficients of the interaction terms were depicted in a heat plot. The descriptive analyses were done in SPSS for Windows, version 25.0. We used packages in R (version 4.0.4; R Foundation for Statistical Computing, Vienna, Austria, 2016; https://www.R-project.org/; RStudio version 1.4.1106) for linear regression, multiple imputation (Mice package, version 3.13.0) and figures (‘forestplot’, version 1.9).

## Results

### Description of general characteristics

Of the 1517 participants, 64.3% were female and the mean age was 56.1 years (s.d. 13.2). Nearly a third of the participants were living alone (29.7%), and 16.5% of the participants had been recently quarantined in the weeks preceding the survey. Switching to teleworking was applicable to 36.9% of our participants, and 16.5% of participants took care of children at home because of school closures (Table 1).

**Table 1:** Characteristics of the study population (*n* = 1517)

Characteristics	
Sociodemographics	
Female gender, *n* (%)	976 (64.3%)
Age, years, mean (s.d.)	56.1 (13.2)
Higher education, *n* (%)	642 (42.3%)
Living alone, *n* (%)	451 (29.7%)
Essential worker, *n* (%)	412 (27.3%)
Clinical, mean (s.d.)	
Number of mental health disorders	2.2 (1.7)
Personality characteristics^a^	
Neuroticism	29.3 (8.4)
Extraversion	31.1 (12.5)
Openness	31.7 (5.5)
Agreeableness	35.3 (11.6)
Conscientiousness	33.3 (11.7)
COVID-19-related, *n* (%)	
Quarantine in prior 2 weeks	250 (16.5%)
Working from home (teleworking)	560 (36.9%)
Taking care of children at home	251 (16.5%)

a. The NEO-Five Factor Inventory was only available for the Netherlands Study of Depression and Anxiety and the Netherlands Study of Depression in Older Persons. The Netherlands Obsessive Compulsive Disorder Association Study used the Five Factor Personality Inventory.

### Main findings

**Fig. 1:**
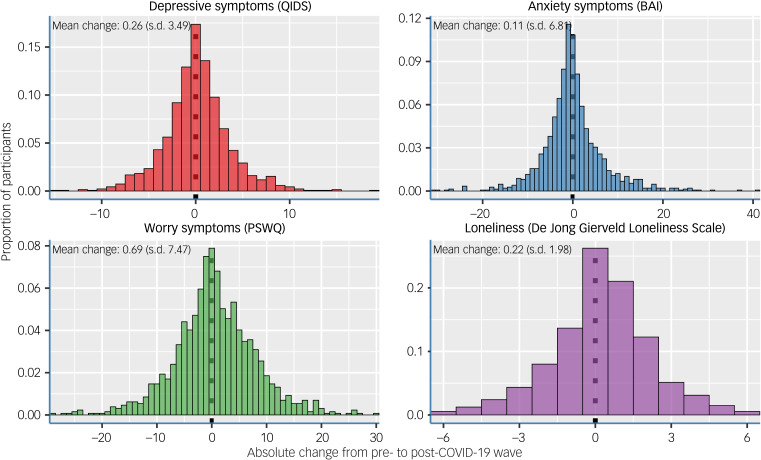
Distribution in histograms of the absolute changes in mental health severity scores from pre- to post-COVID-19 outbreak. The mean changes and s.d. are given per subplot. BAI, Beck Anxiety Inventory; PSWQ, Penn State Worry Questionnaire; QIDS, Quick Inventory of Depressive Symptoms.

We found small mean increases in scores of all four severity scales over time, and substantial variation in the mean changes, as shown in the histograms of Figure 1. The changes over time were significant for three of the four outcomes. The group-level mean values before and during the COVID-19 pandemic were as follows: depressive symptoms (QIDS) changed from a mean of 5.64 (s.e. 0.11) to 6.11 (s.e. 0.12), anxiety symptoms (BAI) changed from a mean of 8.21 (s.e. 0.20) to 8.34 (s.e. 0.24), worry symptoms (PSWQ) changed from a mean of 25.85 (s.e. 0.28) to 27.14 (s.e. 0.29) and loneliness (De Jong Gierveld Loneliness Scale) changed from a mean of 2.04 (s.e. 0.06) to 2.28 (s.e. 0.05). In one sample *t*-tests the changes were as follows: for depressive symptoms (*t* = 2.789, d.f. = 1369, *P* = 0.005, mean change 0.26, 95% CI 0.07–0.45), for anxiety symptoms (*t* = 0.626, d.f. = 1475, *P* = 0.53, mean change 0.11, 95% CI −0.23 to 0.46), for worry symptoms (*t* = 3.324, d.f. = 1292, *P* = 0.0009, mean change 0.69, 95% CI 0.28–1.10) and for loneliness (*t* = 4.021, d.f. = 1287, *P* < 0.0001, mean change 0.22, 95% CI 0.11–0.33).

### Sociodemographic factors

**Fig. 2:**
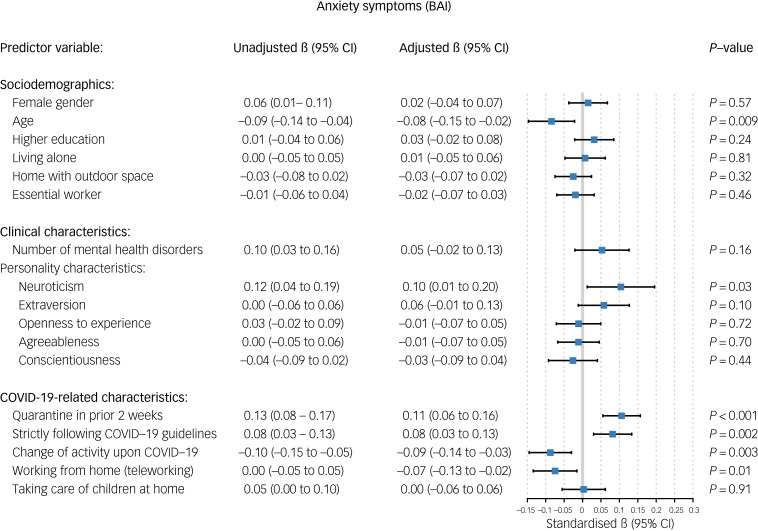
Unadjusted and adjusted associations between sociodemographic, clinical and COVID-19-related characteristics with changes in anxiety symptoms. BAI, Beck Anxiety Inventory.

**Fig. 3:**
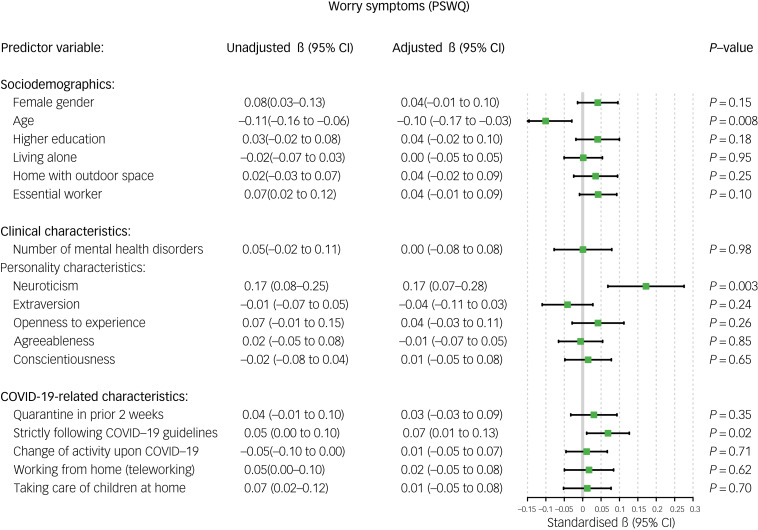
Unadjusted and adjusted associations between sociodemographic, clinical and COVID-19-related characteristics with changes in worry symptoms. PSWQ, Penn State Worry Questionnaire.

**Fig. 4:**
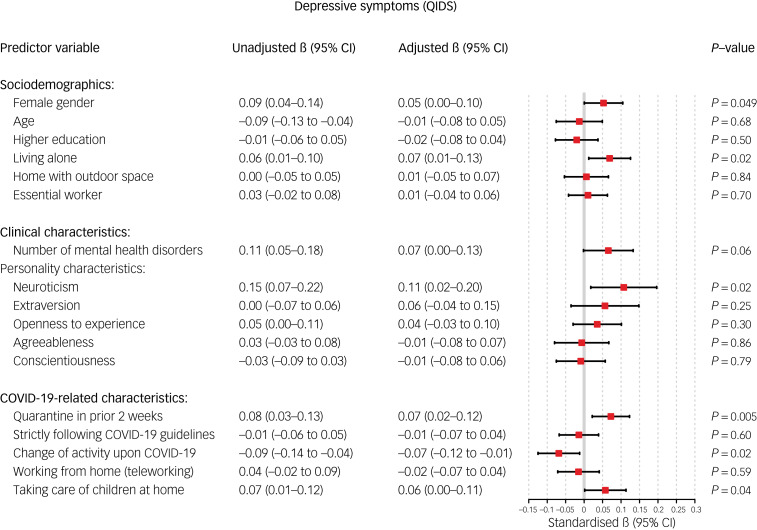
Unadjusted and adjusted associations between sociodemographic, clinical and COVID-19-related characteristics with changes in depressive symptoms. QIDS, Quick Inventory of Depressive Symptoms.

**Fig. 5:**
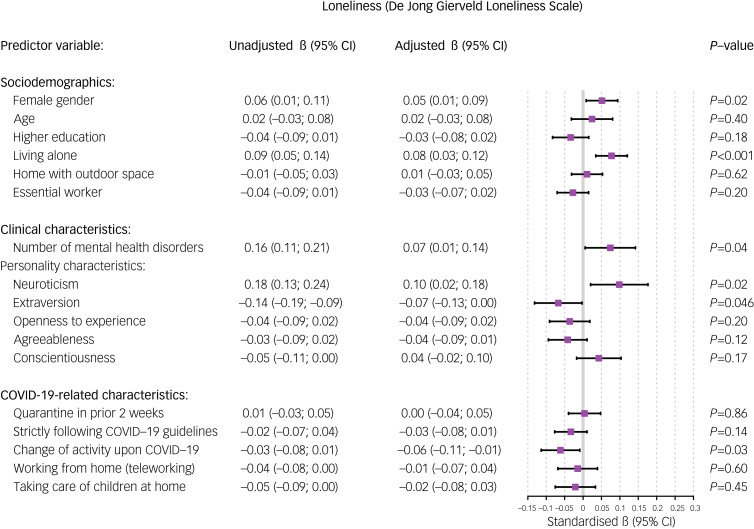
Unadjusted and adjusted associations between sociodemographic, clinical and COVID-19-related characteristics with changes in loneliness (De Jong Gierveld Loneliness Scale).

**Fig. 6:**
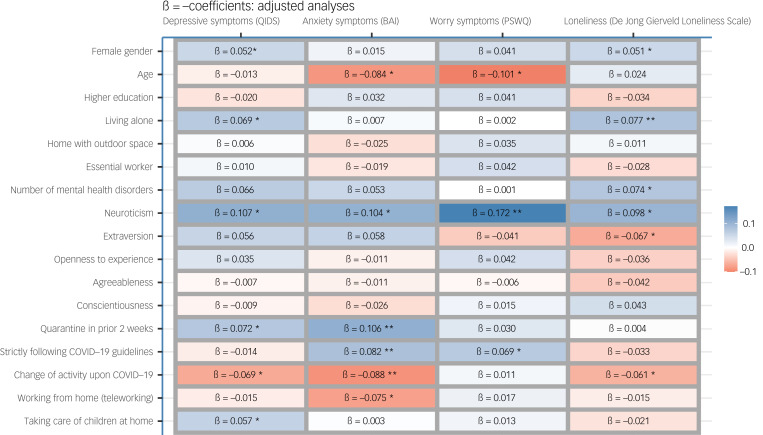
Unadjusted and adjusted associations between sociodemographic, clinical and COVID-19-related characteristics with changes in mental health scores: adjusted analyses. BAI, Beck Anxiety Inventory; PSWQ, Penn State Worry Questionnaire; QIDS, Quick Inventory of Depressive Symptoms.

Older age was associated with a relative decrease in anxiety (standardised β = −0.08, 95% CI −0.15 to −0.02, *P* = 0.009; Fig. 2) and worry symptoms (standardised β = −0.101, 95% CI −0.17 to −0.03, *P* = 0.008; Fig. 3) during the COVID-19 outbreak. Female gender was significantly associated with a relative increase in depressive symptoms (standardised β = 0.052, 95% CI 0.00–0.10, *P* = 0.04; Fig. 4) and loneliness (standardised β = 0.051, 95% CI 0.01–0.09, *P* = 0.02; Fig. 5). Living alone was associated with a higher relative increase in depressive symptoms (standardised β = 0.069, 95% CI 0.01–0.13, *P* = 0.02; Fig. 4) and loneliness (standardised β = 0.077, 95% CI 0.03–0.12, *P* < 0.001; Fig. 5), but not anxiety or worry symptoms. Level of education, the opportunity to go outside or being an essential worker were not associated with changes in mental health (Fig. 6).

### Clinical and personality factors

Higher levels of neuroticism were consistently associated with a higher relative increase in all mental health domains, and especially with higher worry symptoms (standardised β = 0.172, *P* = 0.003; Fig. 6). Additionally, extraversion was inversely associated with loneliness (standardised β = −0.067, *P* = 0.04; Fig. 5). Other personality domains, such as openness, agreeableness and conscientiousness, were not associated with changes in mental health (Fig. 6). A higher number of mental disorders was associated with a relative increase in loneliness (standardised β = 0.074, *P* = 0.04; Fig. 5), but not with any other domains.

### COVID-19-related factors

Regarding COVID-19-related variables, having been in quarantine in the 2 weeks before the survey was associated with a higher increase in depressive (standardised β = 0.072, *P* = 0.005; Fig. 4) and anxiety symptoms (standardised β = 0.106, *P* < 0.001; Fig. 2), whereas strict adherence to COVID-19 guidelines was associated with a relative increase in anxiety (standardised β = 0.082, *P* = 0.002; Fig. 3) and worry symptoms (standardised β = 0.069, *P* = 0.002; Fig. 3). Switching to teleworking was only associated with a relative decrease in anxiety symptoms (standardised β = −0.071, *P* = 0.02; Fig. 2), whereas taking care of children at home was associated with relative increases in depressive symptoms (standardised β = 0.057, *P* = 0.04; Fig. 4). Overall changes in daily activities after the COVID-19 outbreak were associated with relative improvements in mental health domains, except for worry symptoms (Fig. 6).

### Effect modification by burden of mental disorder

Next, potential effect modification by the number of lifetime disorders during the 2006–2016 waves was explored. The interaction terms were generally of low strength, with absolute values of beta-coefficients being <0.05, and of the 68 associations tested (i.e. 17 independent variables×4 outcome variables), only two were statistically significant (2.9%, β-values of 0.06 and 0.08), giving supportive evidence that independent predictors of change in mental health were similar in those with and without a high burden of disorders (Supplementary Figure 1 available at https://doi.org/10.1192/bjo.2022.555).

## Discussion

This study builds on our previous report that showed a modest impact of the COVID-19 pandemic on mental health, although with substantial inter-individual variation.^2^ In the current study, we explored predictors that might explain this variation, and found that neuroticism was the most robust and strongest predictor of mental health deterioration, irrespective of prior mental health burden. This association might be explained by the well-known enhanced stress reactivity and emotion-focused coping strategies used by more neurotic individuals.^13^ Particularly, younger participants were at an increased risk of anxiety and worry. Possible reasons include being more affected by job insecurity, balancing child care and teleworking, and stronger disruptions in daily life because of social distancing and other preventive measures, compared with older participants. Other sociodemographic factors that were relevant for relative increases in depressive symptoms and loneliness included living alone and being a woman. Next, COVID-19-related factors such as quarantine and changes of daily routines were related to relative increases in depressive and anxiety symptoms, whereas strictly following the COVID-19 guidelines was associated with a relative increase in anxiety and worry symptoms. Nevertheless, it is possible that this association between strictly adhering to the guidelines and anxiety/worry symptoms is driven by reverse causation. Persons with higher levels of anxiety and obsessions over contracting infections or spreading germs may tend to engage more in preventive behaviours out of fear. Remarkably, the effect of resilience or vulnerability factors on mental health outcomes was largely similar in those with and without a high burden of psychiatric disorders.

Our findings align with those of previous studies on the impact of quarantine^14^ and personality traits^15^ on how people cope with the COVID-19 pandemic. Previous studies have also shown that not everyone is at similar risk of mental health problems during the COVID-19 pandemic.^3,16^ In the first stages of the COVID-19 pandemic, studies in general population samples identified several sociodemographic risk factors of worse mental health outcomes, such as female gender,^17^ younger age,^18^ lower household income,^19^ living alone,^20^ having pre-school children at home^21^ and higher perceived risk of unemployment.^22^ Specific risk factors related to the COVID-19 pandemic were being quarantined, fear of contagion, having an acquaintance with COVID-19 and a higher exposure to COVID-19-related news.^23–25^ Psychological protective factors included higher levels of emotional stability, self-control, positive coping style and internal locus of control,^26,27^ whereas neuroticism was associated with lower mental well-being.^15^ Interestingly, a longitudinal study conducted during the first 20 weeks of the pandemic in the UK showed in the course of its trajectories that these risk factors still predicted worse mental health outcomes after 20 weeks, despite an observable adaptation to the lockdown effects.^28^

In contrast to previous work that showed an increased prevalence of depression and anxiety among healthcare workers,^29^ we found no association between being an essential worker and changes in mental health. This discrepancy might be attributable to the relatively small percentage of participants who were essential workers in our sample, or the fact that we used a broader definition of essential worker to include other occupations besides healthcare workers, such as being a teacher or police agent.

The main strength of our study is that we replicated previous findings in a well-powered, longitudinal study including long-term pre-pandemic data (gathered through 10 years of follow-up) that allowed us to assess true changes in mental health symptoms that could be attributable to the COVID-19 pandemic. Unlike other studies, we also identified psychiatric status based on several diagnostic interviews and included personality traits based on validated questionnaires. Notably, longitudinal studies on the impact of personality traits on mental health during the COVID-19 pandemic are still scarce, and often do not include pre-COVID-19 pandemic assessments.^15^ Other strengths of our study are the wide variability in changes in symptom severity, a wide age range (30–90 years) and the inclusion of both participants with and without a history of affective disorders; therefore, our findings likely also apply outside the context of this study.

It is important to note that our findings only apply to the first 2 months of the outbreak and might not reflect the long-term effects of the COVID-19 pandemic, which may be different. It is also relevant to place the study findings in light of the context and course of the pandemic in The Netherlands, a small country in Western Europe, limiting the generalisability of our results to countries with different sociodemographic or socioeconomic circumstances. The Dutch Government announced the first lockdown measures in March 2020, when daily infection rates started to increase. Day-care centres, schools, cinemas and most shops were closed, and the population was instructed to stay at home and avoid contact with those who were not members of their household. Teleworking was recommended and social distancing measures (e.g. no public events or private parties) were implemented. Temporarily, there was also a night curfew, although this measure was implemented after the span of data collection of the current study. This nationwide lockdown lasted from mid-March to early May 2020, and severely restricted people's lives in an unprecedented manner. This lockdown was followed by a policy of relaxations from early May to early July 2020. During the relaxation period, shops reopened, and day-care centres and schools partially reopened. It is possible that the findings would be different during this relaxation period, as more social contacts were allowed.

Moreover, we would like to highlight that participants chose actively to participate in the COVID-19 online questionnaire and the response rate was rather low (58%), which may have introduced some selection bias. It is possible that respondents were health-conscious individuals and more concerned about the outbreak. Non-respondents were more likely to have a pre-existing mental health disorder, which could affect our findings by underestimating the mental health impact on individuals with mental health disorders. Finally, we used different assessment methods (face to face versus online) for the pre-pandemic and post-pandemic assessments, respectively. However, this difference applied to all respondents, and thereby is unlikely to have affected differential associations with predictors.

Our findings have some implications for public health. First, it can lead to identification of at-risk groups as well as more personalised psychological or psychiatric treatments based on a patient's individual profile. Since mental health is likely to remain a key issue in the post-COVID-19 period,^30^ it is crucial to identify the individuals that have suffered greater declines in their mental health and, more generally, to shed further light on what works for improving mental health. We know, for instance, that persons with high neuroticism are less prone to follow preventive guidelines during the COVID-19 pandemic,^3^ especially in the case of comorbid depression.^4^ One explanation is that persons with high neuroticism may tend to dissociate from threatening information as a defence mechanism to reduce their underlying fears.^5^ Irrespective of the potential mechanisms, in times of pandemic, neuroticism should be considered an important vulnerability factor for deterioration of mental health. Second, it can highlight the unintended consequences of COVID-19 restrictions. Lockdowns were effective in bringing case numbers down to a more manageable level and easing the pressure on health systems, but they also came with significant adverse effects on mental health. Identifying groups at risk only based on sociodemographic information may overlook people in need of help, such as individuals with higher neuroticism.^31^ Based on our findings, it would be helpful to develop communication strategies that offer clear information on the pandemic, to engage people with higher neuroticism in precautionary behaviors.^6^ Furthermore, early detection could be helpful for people with multiple risk factors, including high neuroticism, younger age, female gender, living alone and being quarantined. Interventions such as telemedicine, self-monitoring, stepped care and prevention of loneliness are important ingredients for both acute crisis management and more routine support and care.

In conclusion, our study provides unique information on the longitudinal variability of mental health during the COVID-19 pandemic, and insights into the factors that contribute to resilience or vulnerability in stressful situations.

## Data Availability

According to European law (General Data Protection Regulation), data containing potentially identifying or sensitive patient information are restricted. However, for academic researchers, data can be made available on request via the NESDA (nesda@ggzingeest.nl), NESDO (d.rhebergen@ggzcentraal.nl) and NOCDA (p.vanoppen@ggzingeest.nl) data access committees.
